# Gene expression models based on a reference laboratory strain are poor predictors of *Mycobacterium tuberculosis* complex transcriptional diversity

**DOI:** 10.1038/s41598-018-22237-5

**Published:** 2018-02-28

**Authors:** Álvaro Chiner-Oms, Fernando González-Candelas, Iñaki Comas

**Affiliations:** 1 Unidad Mixta “Infección y Salud Pública” FISABIO-CSISP/Universidad de Valencia, Instituto de Biología Integrativa de Sistemas, Valencia, Spain; 2 CIBER en Epidemiología y Salud Pública, Valencia, Spain; 3 Instituto de Biomedicina, IBV-CSIC, Valencia, Spain

## Abstract

Every year, species of the *Mycobacterium tuberculosis* complex (MTBC) kill more people than any other infectious disease caused by a single agent. As a consequence of its global distribution and parallel evolution with the human host the bacteria is not genetically homogeneous. The observed genetic heterogeneity has relevance at different phenotypic levels, from gene expression to epidemiological dynamics. However, current systems biology datasets have focused on the laboratory reference strain H37Rv. By using large expression datasets testing the role of almost two hundred transcription factors, we have constructed computational models to grab the expression dynamics of *Mycobacterium tuberculosis* H37Rv genes. However, we have found that many of those transcription factors are deleted or likely dysfunctional across strains of the MTBC. As a result, we failed to predict expression changes in strains with a different genetic background when compared with experimental data. These results highlight the importance of designing systems biology approaches that take into account the genetic diversity of tubercle bacilli, or any other pathogen, if we want to identify universal targets for vaccines, diagnostics and treatments.

## Introduction

Tuberculosis has been a scourge to humankind for millennia^1^. Today, TB is the leading cause of death by a single infectious agent worldwide^2^. In humans, the disease is caused by *Mycobacterium tuberculosis* and *Mycobacterium africanum*, which belong to the *Mycobacterium tuberculosis* complex (MTBC), along with other species that cause the disease in animals. The bacterium infects the host through the respiratory tract. Once in the lungs, it is phagocyted by macrophages, which typically are encapsulated in a granuloma^3^. The bacteria can be dormant and survive inside the granuloma during months, years or even decades in an asymptomatic disease state called latency^4^. The transition from latency to an active disease state depends on biological features of the bacteria, the host, environmental factors and the interactions among all of them^5^. These interactions are not completely understood yet^6^. Moreover, animal models are widely used but they do not reproduce perfectly the human-pathogen interaction^3,7^.

One way to approach the complexity of the host-pathogen-environment triangle is through systems biology. In the case of TB, systems biology approaches have produced encouraging results in the identification of persistence genes^8^, the pharmacokinetics and pharmacodynamics of TB drugs inside the granuloma^9,10^, and the identification of drug resistance mechanisms^11^. Overexpression experiments and chromatin-immune-precipitation sequencing (ChIP-Seq) data have been used to produce a detailed map of the interactions and regulatory logics of more than 200 transcription factors (TFs) in H37Rv, the laboratory reference strain^12,13^. The enormous amount of data generated is publicly available and can be used to study the regulatory interactions of the bacteria in several ways^14^.

However, little attention has been paid to the fact that H37Rv is a clinical strain used in laboratories for decades and that in many aspects it does not represent the whole species. Therefore, natural perturbations in the biological networks inferred in H37Rv, introduced by naturally occurring mutations in clinical strains, will likely change the gene model architecture and predictions and the underlying regulatory network. In fact, through their evolutionary history, *M*. *tuberculosis* complex strains found in humans have diverged in 7 different lineages^15^. The aforementioned H37Rv strain belongs to lineage 4. Most of the genetic variation among lineages in *M*. *tuberculosis* results from wide genomic deletions and point mutations^16^. It is also known that the maximum genetic distance between strains of different lineages is around 2,500 SNPs^17^. The phenotypic role of mutations defining lineages has been extensively studied and some of them are clearly linked to transcriptional differences between the MTBC lineages^18–20^. It is also clear that one single mutation affecting regulatory processes can impact dramatically on the virulence of the pathogen^21,22^. In fact, a novel live vaccine, attenuated by carrying a deletion in the key regulator PhoP, is currently in phase 1B of clinical trials^23^. In addition, several studies have shown that bacterial genetic diversity has epidemiological implications and genetic differences among lineages lead to differences in the immune response and disease progression in the host^16,17,24–26^. As a result, novel diagnostics, vaccines and treatments may be compromised by failing to account for the circulating diversity as recently described for several diagnostics tests based on the detection of the protein of Mpt64^27^.

Thus, we are completely blind on whether the topology of the regulatory networks and the gene expression mathematical models derived from H37Rv can be extrapolated to other strains of the MTBC and on how the regulatory modulations are affected by the existing bacterial diversity. In this work, we derive new gene expression models by pooling existing H37Rv data and explore their predictive power on genome-wide expression patterns when natural variations (mutations) found in clinical strains are considered. We show how different experimental setups can affect the inferred models of gene expression and regulation and how far we are from predicting, only from transcriptomic data, the impact of genetic polymorphisms at a genome-wide expression level.

## Results

### Building and validation of gene expression models based on strain H37Rv, lineage 4

By taking advantage of recently published experimental data sets testing the regulatory influences of known TFs, we defined gene expression models for the laboratory reference strain H37Rv. The data sets included transcription factors overexpression experiments (TFOE) for ~200 TFs (~700 tiling microarray experimental tests)^12^. Our aim was to model, for each gene, the level of expression and the resulting changes therein as a function of varying the expression of each TF. We built the models using a linear regression approach as described^28^.

**Figure 1:**
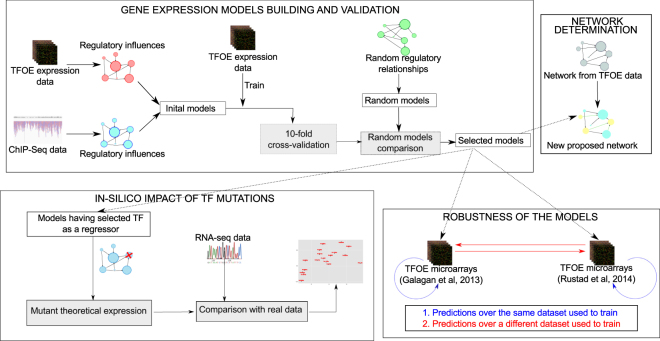
Workflow to build and validate gene expression computational models. Initial models were derived from the regulatory relationships derived from the TFOE and ChIP-Seq data. They were trained with the data set from Rustad *et al*.^12^ (see Materials and Methods for more details about the different data sets used). These initial models went through a 10-fold cross-validation process, to discard low accuracy models. The remaining ones were compared with random models. Those showing better performance than the random models were selected as final models. These models were used to (i) cross-check the robustness of the models, (ii) derive a new regulatory network, and (iii) predict the impact of a TF deletion *in silico*.

**Figure 2:**
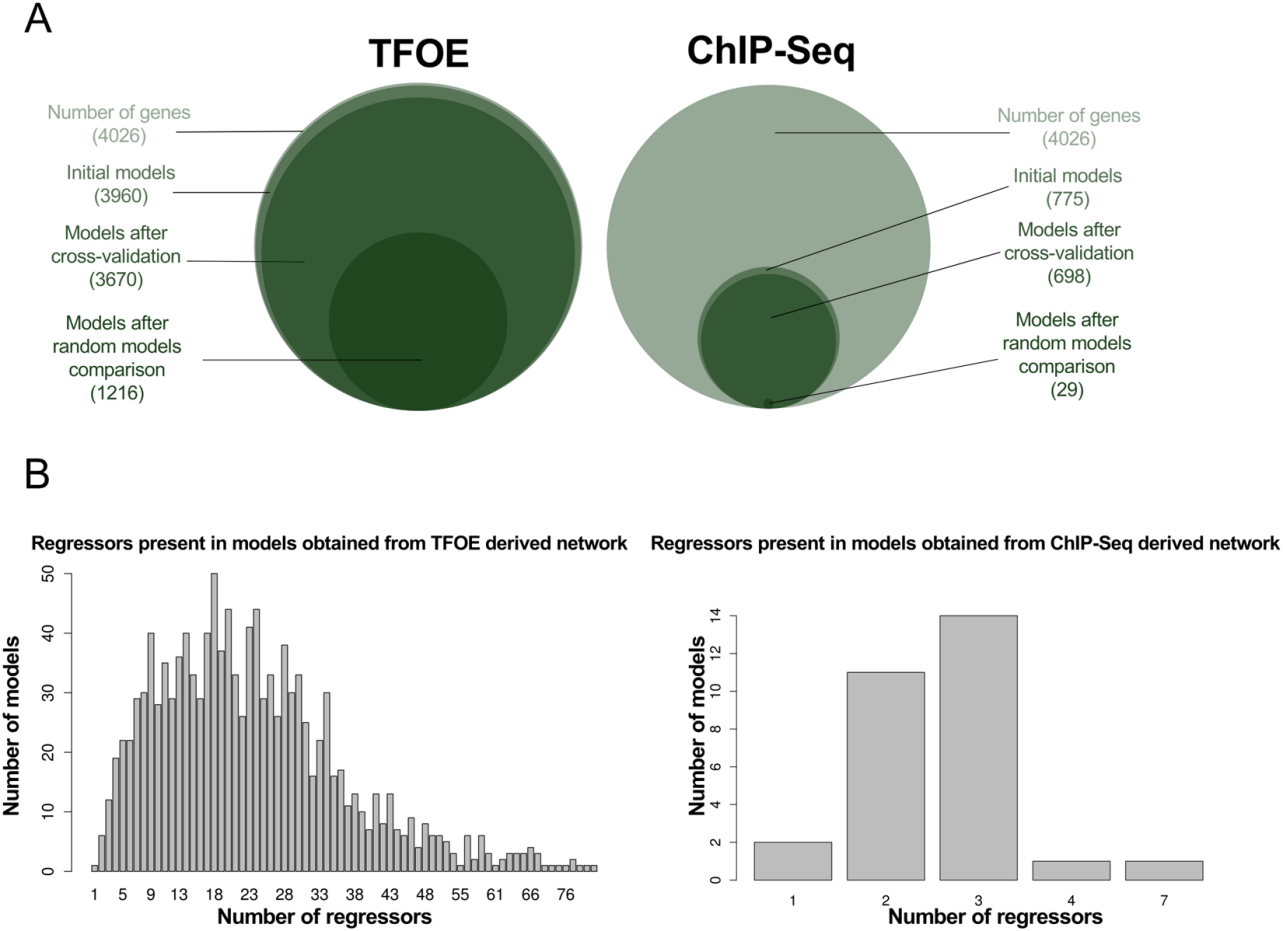
Gene expression computational models. (**A**) Overview of the results obtained during the building process and refining steps of the computational models derived from the TFOE data (left) and the ChIP-Seq data (right). (**B**) Distribution of the number of TFs affecting the target gene on each network model.

Using a backward stepwise algorithm (Fig. 1, see Material and Methods for details), we generated 3,960 putative gene expression models. When, in addition, we required evidence of physical interaction from CHIP-seq data, the number of initial models was reduced to 755. Therefore, our putative gene models accounted for 98.3% of the coding capacity of the genome when physical interaction was not required, and only 19.24% of it when we used the ChIP-Seq data. Secondly, we cross-validated all the models in the two data sets and then compared them with random models to discard spurious results. Following this approach, we discarded 2,744 models for the TFOE and retained 1,216 (30.8%). For the case of ChIP-Seq data, only 29 models were retained (3.74% of the initial models) (Fig. 2A). The models derived from TFOEs alone included a larger number of TFs (regressors) per model, as expected due to the larger number of regulatory events incorporated. On the contrary, the models derived from the combination of TFOEs and ChIP-Seq data had fewer TFs influencing the expression, as they only include those TFs physically bound to the gene (Fig. 2B). In summary, our approach shows the relevance of performing sequential statistical validations of expression models derived from experimental data.

**Figure 3:**
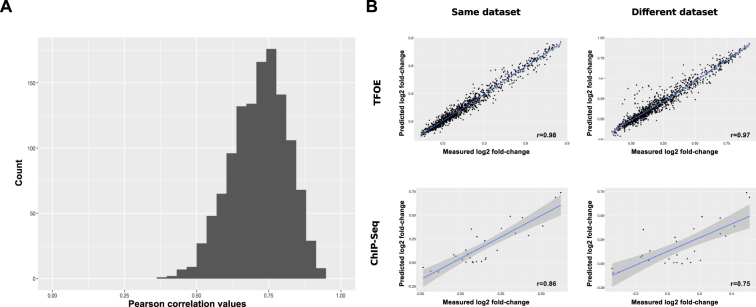
Comparison of models performance over different data sets. (**A**) Distribution of the Pearson correlation values obtained when comparing each gene expression measure with its predicted expression. (**B**) There is one data point for each model. Each dot in a plot is a measure of the gene expression fold-change between Rv0001 and the gene represented by this model. The y-axis corresponds to the predicted fold-change in gene expression while the x-axis corresponds to the measured fold-change. The upper row refers to the models derived from the TFOE data set. The lower row contains the models derived from the ChIP-Seq data set. The values in the left column were calculated when training and predictions were performed with the same data set^12^. The values in the right column were calculated when different data sets were used for training and predicting.

There is a limited agreement between the values predicted by the TFOE-derived models and the observed values (average of the Pearson correlation coefficients = 0.71) (Fig. 3A) despite being trained by the same dataset. To evaluate how robust the predictions were to experimental noise, we compared them with the expression values obtained in a previous, analogous TFOE experiment^28^. To compare the predictive power of the models across data sets we used the housekeeping gene Rv0001 (*dnaA*) as a reference for the expression values. We measured the average fold-change in expression values between *dnaA* and the remaining genes across all the samples. When we compared the fold-change with the observed Rustad data set, we found a correlation coefficient of 0.98 (p-value < 0.01) (Fig. 3B). When we compared the predicted fold-change with the Galagan data set, the correlation coefficient was 0.97 (p-value < 0.01). As expected, in the first case the correlation was almost perfect, because the models are making predictions over the same data set used to calculate their regressors’ coefficients. However, predictions over a similar but different data set were less accurate but still a high predictive power was achieved.

Having established that gene expression models can predict gene expression trends in TFOEs experiments, we tried to predict absolute expression values in the Galagan data set^28^. We correctly predicted the expression for only 128 genes (10.52% of TFOEs-derived gene models, pFDR ≤ 0.01). In fact, the comparison of average expression values for each gene between the two datasets (Galagan vs Rustad) revealed that only in 18 cases the mean expression values for the same gene were not different (pFDR < 0.01, see Supplementary Fig. S1 for more information). Taken together these results show that experimental noise across laboratories has a large influence on the results for analogous experiments, at least for the prediction of absolute quantitative expression levels.

### Regulatory network based on statistically validated interactions

**Figure 4:**
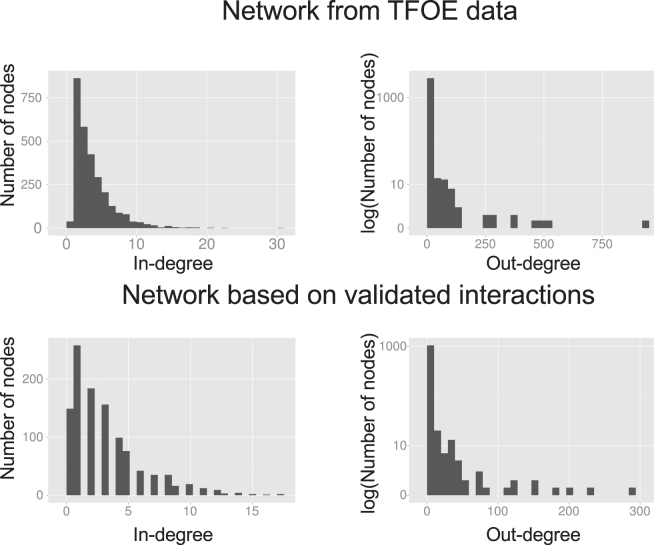
Validated gene expression network. Comparison of the out- and in-degree distributions between the network derived from TFOE data and the network derived in this study.

The 1,216 expression models obtained from the TFOE data set included 11,253 regulatory relationships. These relationships are the ones selected after applying the backward step-wise method in the building process of the models (see Material and Methods for details). Although all of them led to a lower Bayesian Information Criterion in their respective models, most of these relationships are based on a weak regulatory signal. To select the strongest links between TFs and gene regulation influence, we kept those leading to a significant change in gene expression (two-fold change) according to TFOE data^12^. We built a new regulatory network with these subsets of regulatory relationships. The new network comprised 3,396 regulatory events across 1,102 genes (37.15% of the events and 38.76% of the genes from the network proposed by Rustad *et al*.^12^). The distribution of the in-degree parameter of the network (Fig. 4) revealed that most genes are regulated by an intermediate number of factors whereas a minority is regulated by a large or small number of them. On the other hand, the distribution of the out-degree parameter followed the expected power-law distribution^29^, with most TFs regulating a small amount of genes and a few genes affecting the regulation of many (see Supplementary Table S1 for a more details).

In agreement with the original networks of Rustad *et al*. and Galagan *et al*., in this new regulatory network Rv0023 and Rv0081 are the TFs that regulate the largest number of genes (672 and 627, respectively). Thus, these genes are regulatory hubs of *M*. *tuberculosis*. On the other hand, Rv3202c is the gene with the largest number of TFs influencing its expression, as it is indirectly regulated by 26 TFs. This gene has ATPase and helicase activities^30^. The regulatory subnetwork of Rv3202c is related to regulatory DNA and RNA processes as well as to response to external stimuli, transport and secretion. The new network derived can be found in Supplementary Table S2.

### Transcription factors are not universally conserved in the MTBC

Once gene expression models and a regulatory network for H37Rv were available, we tried to predict the phenotypic effect of natural genetic variation observed in circulating clinical strains. For this, we first examined the degree of conservation of the studied TFs across the *Mycobacterium tuberculosis* complex. Previous studies have identified mutations in the genes that code for the PhoPR system in MTBC strains that had important effects on the pathogen’s virulence^21^, so that not only SNPs in the regulatory regions of the TF but also those located in the coding region could lead to differences in TF activity. Thus, we focused our analyses on mutations falling in regulatory regions but also on those coding mutations that might impair the normal function of the TF.

**Figure 5:**
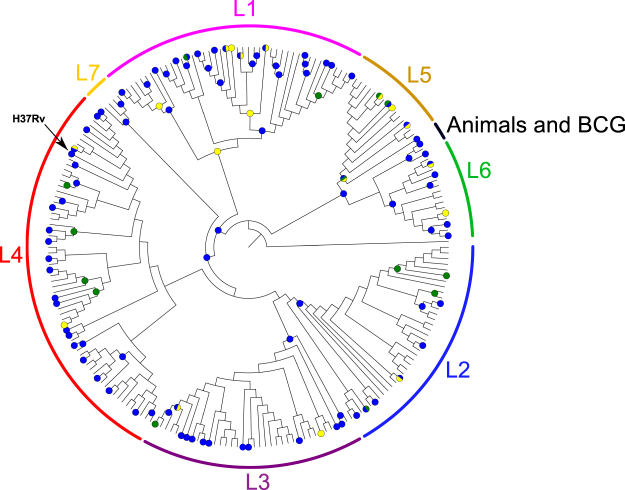
MTBC phylogeny comprising the seven major lineages. The figure represents the number of TFs missing or potentially affected in their regulatory functions in one or more clinical strains from the Comas *et al*.^15^ MTBC reference dataset (n = 219 strains). Mutations affecting a TF are mapped to the corresponding internal/external node of the Comas *et al*. phylogeny and highlighted in colour. Green colour indicates total or partial deletions of a TF, yellow indicates stop-codon gain or loss, and blue indicates a SNP in the regulatory region of a TF. The maximum-likelihood phylogeny was obtained from a genome-wide SNP alignment as described in Comas *et al*.^15^.

**Figure 6:**
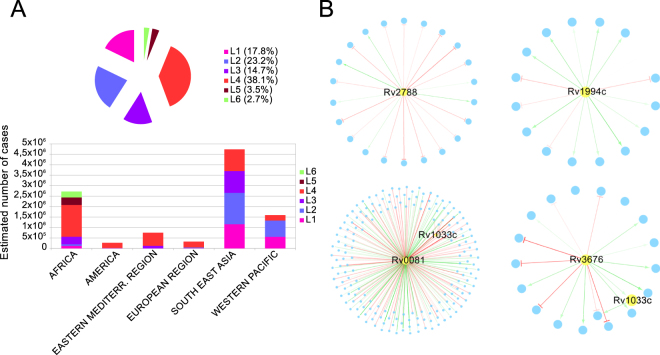
Global incidence of the different lineages and representative examples of mutations affecting complete lineages. (**A**) Pie chart showing the estimated number of annual tuberculosis cases attributed to each lineage and a barplot showing the incidence of the different lineages by region. Lineage 7 is not shown due to its low incidence in global terms. The data related to the disease incidence by region come from the WHO^2^ and the lineage abundance for each region from a previous work^16^. (**B**) Examples of regulatory subnetworks of transcription factors affected by mutations in one or more lineages. From upper-left to lower-right: regulatory subnetwork of Rv2788 (early stop-codon in all lineage 1 strains); regulatory subnetwork of Rv1994c (deleted in all lineage 5 strains); regulatory sub-network of Rv0081 (SNP in regulatory region found in all the strains screened from lineage 2,3,4 and 7) and regulatory sub-network of Rv3676 (SNP in regulatory region in all the strains from lineage 3). Only TF (yellow nodes) were labeled. Green edges indicate positive regulations whereas red edges indicate negative regulation. The intensity of the edges is related to the influence of the TF → on the gene (the darker the edge, the higher the regulatory effect).

Using a collection of genome-wide SNPs and indels from a large number of clinical strains^15^, we identified a total of 28 transcription factors (TFs), among those present in the TFOE data^12^, that are missing or likely dysfunctional (as defined in Material and Methods) in one or more clinical strains, including 4 affecting complete lineages of the *M*. *tuberculosis* complex (Fig. 5). Some of these transcription factors are missing in complete lineages and sublineages as they are in known regions of difference (RD) used as phylogenetic markers^16^ (all the deletions detected shown in Supplementary Table S3). For example, Rv1994c and Rv2478c are in RD743 and RD715 and they affect the entire lineage 5^31^. Those lineages represent up to 50% of tuberculosis cases in West Africa^32^. We have also identified single point mutations disrupting the normal functioning of some TFs (Fig. 5 and Supplementary Table S4). This is the case of *sirR* (Rv2788). An early stop codon mutation was found in all the strains of lineage 1. In the proposed regulatory network, Rv2788 regulates 22 genes (Fig. 6B) and, accordingly, 16 of those genes were expressed differentially in lineage 1 strains with respect to H37Rv using RNAseq data^18^. In our estimates (Fig. 6A), lineage 1 accounts for roughly 18% of the strains causing active tuberculosis cases each year (almost 1.9 million cases/year). In light of the existing variation in TFs and other regulatory elements among clinical MTBC strains, it is very important to take the circulating diversity when building comprehensive regulatory networks, as these may differ among strains with different variants.

Next, in order to identify the main biological processes involved, we analyzed the relative abundance of Gene Ontology (GO) terms in the regulatory subnetworks for each affected TF. Most of the TFs identified as missing in clinical strains have an important role, with a direct or indirect regulatory influence in up to 210 genes. The GO analysis showed that a wide range of processes are significantly overrepresented among affected TFs, including specific metabolic, regulation, pathogenicity and response to external stimuli pathways (Supplementary Table S3 and Supplementary Table S4). Some deletions affecting TFs appear in single strains, such as one affecting Rv1994c in a strain of lineage 2 or Rv1776c in a strain of lineage 3. A deletion of gene Rv1985c, a known antigen, was also found in a group of strains belonging to lineage 1. It is also remarkable that a stop-codon gain mutation was found in Rv0465c (also known as *ramB*) in one strain of lineage 4. RamB is related to the glyoxylate cycle in the pathogen and it has been proposed to play an important role in the adaptive response of the bacteria to different host environments during infection^33^. Moreover, the regulatory subnetwork of *ramB* is involved in several processes such as regulation of RNA biosynthesis, response to hypoxia or interaction with the host.

We also identified 117 SNPs located in the regulatory regions of 44 TFs (Fig. 5, Supplementary Table S5). Most of these SNPs affect primary or alternative transcription start sites (TSS), as defined previously^34^; two of them correspond to antisense TSS and two more were internal TSS. Seventy-four of these SNPs affect one single strain, with the remaining 43 affecting more than one strain. Interestingly, only a few of them affect complete lineages, such as T89200G, which impacts the master regulator Rv0081 in modern lineages 2, 3, 4 and 7 (76% of the circulating strains), or C422745T, which impacts Rv0353 in all lineages except 5 and 6. Rv0081 regulates 188 genes (including *tcrR*, which also regulates 26 genes) (Fig. 6B). Hence, a SNP potentially affecting Rv0081 regulation could have an important effect on the regulatory network of the bacteria^21^. Besides, we found one homoplastic SNP (C2965900T, which affects Rv2642) that has emerged independently in strains of three different lineages. It has been shown previously that some of the SNPs screened affect the expression of their corresponding TF. For example, SNP G3500149A has been reported to be involved in the regulation of TF Rv3133c in Beijing strains (lineage 2), as it creates a –10-box leading to the overexpression of the DosR regulon^18,35^. The Supplementary Fig. S2 includes a detailed view of the phylogeny with all the variants marked on it.

### *In-silico* expression prediction of genetic backgrounds observed in a clinical and in a vaccine strain

To explore how well the H37Rv-based expression models and the validated network predicted the impact of the genetic background in the transcriptional landscape of the bacteria we selected a lineage 1 strain (T83) from the comparative genomics analysis. For T83 there is publicly available expression data set^18^ and we have identified a deletion in TF Rv1985c and an early stop-codon in TF Rv2788. Rv1985c or Rv2788 are present in 169 gene models. By reducing the expression of Rv1985c and Rv2788 to its minimum level, we created gene models mimicking the T83 genetic background. With these modifications, we were able to predict that 148 genes will have a significant change in their absolute expression value (pFDR < 0.05). To formally compare with experimental data, we used the RNA-seq data sets from H37Rv and T83. Of those 148 genes only 71 changed in the same direction as determined in RNA-seq dataset irrespective of the absolute expression value (Supplementary Table S6). Moreover, out of the 148 genes only 64 showed differential gene expression in RNA-seq experiments with the same strain and with no correlation between predicted and observed values (Pearson correlation coefficient = 0.08, p-value = 0.48) (Supplementary Fig. S3A). Although conclusions from a single strain are necessarily provisional, it is also true that mutations in T83 are present in several strains of lineage 1. Thus, from the limited data available we speculate that gene expression models based on H37Rv and derived from TFOE are not likely to predict accurately enough the transcriptional landscape of *M*. *tuberculosis* complex lineages.

We reasoned that, given that it is not possible to predict expression changes in different genetic backgrounds, gene expression models might still be valid for experiments using the H37Rv background. As an example, we selected PhoP for several reasons: (i) it is one of the main regulators in the MTBC^22,28^; (ii) it is the main gene deleted in a vaccine candidate that is already in clinical trial phase 1B^23^; (iii) there are large data sets available on the expression changes in knock-out strains using two different approaches, microarray^36^ and RNAseq^37^; and (iv) there is strong evidence that mutations in the PhoPR regulatory regions impact fitness of clinical strains in the human host^21^.

From the TFOE, we identified 218 models in which *phoP* (Rv0757) is present as a regressor. We lowered the expression value of *phoP* in the models to the minimum, thus simulating that the gene is knocked-out. Comparing the simulated knock-out with the wild-type models, we detected 188 genes with a statistically significant difference in expression (pFDR < 0.05). Very little overlap was found between the 188 genes predicted and those found experimentally to be impacted by a knock-out mutant. Of the 188 predicted genes, Gonzalo-Asensio *et al*.^36^ described only 10 in microarray experiments. The predictions for these genes can be found in Supplementary Table S6. We also contrasted our predictions with an RNA-seq data set of a *phoP* knockout H37Rv strain^37^. We first compared whether the predicted expression for the 188 genes followed the same direction as the ones from the RNA-seq data set. In 96 cases the predictions agreed with the experimental values but in 92 cases the predictions failed (Supplementary Table S6). Cohen’s kappa test showed a slight agreement between the real and the predicted values (kappa = 0.05). Next, we compared the 188 predictive models showing differential expression with the genes showing differential expression in the data set (adjusted p-value < 0.05) and we found only 9 coincidences. For these 9 genes, we observed no statistically significant correlation (Pearson correlation coefficient = 0.59, p-value = 0.09) (Supplementary Fig. S3B) between the predicted and measured gene expression fold-change in the mutant.

**Figure 7:**
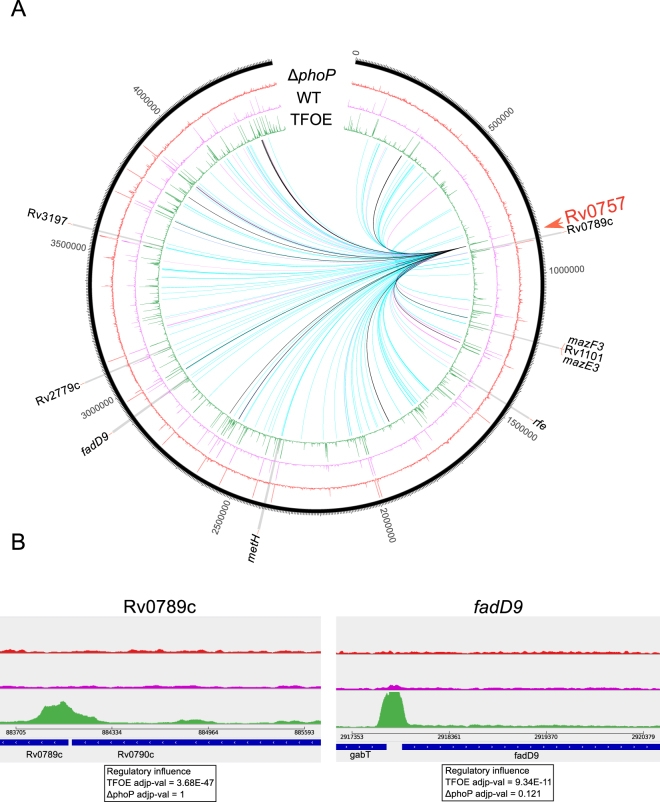
ChIP-Seq coverage comparison and regulatory influences between the *phoP* knock-out, wild-type and *phoP* overexpressed strains. (**A**) Circle representation of the H37Rv reference genome. From outside to inside: ChIP-Seq coverage of *phoP* knock-out mutant, wild-type and TFOE. The inner links represent the regulatory influence of *phoP* derived from TFOE (blue), mutant strain (purple) and their overlap (black). (**B**) Detail of two selected genes with regulatory influences derived from TFOE that do not match the evidence from WT and the mutant strain.

We tested whether the lack of correspondence between our predictions and the experimental data might be due to the former being obtained from TFOE whereas the later were defined after analyzing a knock-out strain. Figure 7A shows a graphical comparison between the ChIP-Seq coverage of the over-expressed, the knock-out mutant, and the wild-type strains. Using the wild-type coverage vs the *phoP* mutant coverage as a negative control, we were able to infer the binding sites of PhoP in the H37Rv strain^37^. By comparing these results with the binding sites inferred from the TFOE strains, we observed differences in several genes (the 9 genes showing differences in ChIP-Seq coverage are highlighted in Fig. 7A). Details for two of these genes are shown in Fig. 7B and more examples are included in Supplementary Fig. S4. For example, for the Rv0789c gene there is no evidence of PhoP regulation when the mutant and the wild-type are compared. However, a peak appears in the overexpressed strain and strong regulatory evidence has been reported^12^. In total, from 139 genes predicted to be regulated by PhoP from the TFOE data and 51 from the mutant data, only 16 genes overlapped. Thus, different methodologies to test the function of a gene (overexpression versus deletion) partially account for the limited predictive power of the H37Rv gene expression models even when the mutant is derived from a H37Rv background.

## Discussion

Systems biology approaches rarely accommodate information about natural polymorphisms in the systems studied. In addition, even for model organisms, systems biology approaches has been rarely applied to more than one genetic lineage^38^. Thus, the actual power of systems biology models in predicting the effect of new mutations on non-model systems still has to be evaluated^39^. This is the case for tuberculosis, in which both the pathogen and the host are not genetically homogeneous and genetic variation in any of them may impact disease progression and outcome^40^.

We have tested the predictive power of state-of-the-art *M*. *tuberculosis* regulatory networks and expression models when the system is disturbed by (i) experimental noise, (ii) mutations associated to a clinical strain with a different genetic background to that of the training data set, and (iii) a knock-out mutation in the key regulator PhoP in the reference strain used for the training data set. For the genetic background and single mutations predictions our results show very little overlap between the genes predicted to be significantly impacted and those determined experimentally. Overall, these results suggest that our predictive models only grab a minor part of the true phenotypic variation.

One striking result is that gene expression models are not statistically different from random generated models in 66.87% of the cases. This result suggests that subtle impacts of TFs in expression maybe missing even for a data set that comprises the construction of 206 TFOEs strains and 698 microarray for the analysis. In this analysis, only the TF having a strong signal were taken into account. Background noise introduced by the experimental system prevented us from incorporating TFs with more subtle effects. Thus, we cannot discard the possibility that the sum of weak effects may account for some of the expression differences. For the remaining models there is a moderate correlation with predicted absolute quantitative expression (r = 0.71) and a high correlation (r = 0.98) to predicted fold-changes using *dnaA* to normalize. When applied to an analogous data set we found a good agreement with fold-change data (r = 0.93) but almost none when we tried to predict absolute expression values (128 out of 1216). Thus, although the models grab the intensity of the interaction they are not able of quantifying the changes.

The limitations of the models, even when applied to the same data used to train them, can be explained by two different, non-mutually excluding alternatives.

Firstly, our results show that the statistical validation of gene expression models is essential to remove methodology-dependent effects that may or may not correspond to actual biological differences and contrasted biological effects. Only 1,216 gene models derived from the TFOE dataset and 29 from the ChIP-Seq were significantly different from random-generated gene expression models. In addition, predictions with an alternative data set, generated in a different laboratory but following the same protocols, show that absolute quantification of gene expression is not possible with current models. This suggests that, in order to understand the impact of different perturbations in the system such as genetic mutations, the noise introduced by the experimental setting must be taken into account, especially in genes with a low expression level^41^.

Secondly, the regulatory network inferred is highly dependent on the experimental methodology. Overexpression of transcription factors is a common, widely used technique to identify regulatory influences but it can fail in making accurate predictions when an increase in gene expression has no physiological effect or, on the contrary, it can overestimate the regulatory effect due to a loss of specificity^42^. A recent work with *Mycoplasma pneumoniae* demonstrated that the overexpression of regulatory molecules (asRNAs in this case) leads to an overestimation of the regulatory effect of these molecules^43^. In addition, the ChIP-Seq technique might introduce false positives when overexpressing the TF^44^. Besides, other studies^45^ have shown that there are many sRNAs in bacteria that regulate gene expression. Their effect is not reflected in this type of networks and analyses, as we only look for the amount of mRNA produced by a gene. Finally, the amount of mRNA does not always agree with the amount of translated protein^46,47^. However, new experimental techniques, such as CRISPRi, are being tested in *M*. *tuberculosis* and other organisms to characterize and modify gene expression^48^.

Our results show that the different TFs tested in H37Rv are not universally conserved. Some of those mutations (deletions and single point mutations) are present in complete lineages and in up to 76% of the circulating strains. Using comparative genomic data we have predicted the transcriptional landscape of a lineage 1 strain. We found 64 out of 148 matches between the genes predicted to be impacted and those found in an RNA-seq experiments^18^. Strain T83 belongs to lineage 1 and its genetic distance to H37Rv, the strain used to build the models, is more than 1,800 SNPs^15^. Thus, other genetic differences besides those found in TFs between this strain and the one used to infer the regulatory influences will certainly impact the genome-wide transcriptional landscape of T83. For example, we have mapped two SNPs in the regulatory region of TF Rv0353 in T83. Current models do not take into account the potential influence of these SNPs nor that of other regulatory layers that possibly differ between lineages. In addition, we have previously shown that specific SNPs of lineage 1 alter the expression levels of sense and antisense transcripts by means of new TSSs^18^. Our predictions on a PhoP knock-out H37Rv strain were also poor. The regulatory influence was predicted correctly only for 96 out of 188 genes.

The limitations of current models do not preclude that significant results can be obtained from their application^11^. However, it is apparent that at least lineage- and condition-specific models will be necessary to generate more accurate predictions across the *Mycobacterium tuberculosis* complex. We are aware that meeting the above conditions is a major experimental and computational accomplishment. For example, the data used in this work were derived from almost 200 TFs genetic constructs and ~700 microarray experiments and used only strain H37Rv. Generating comprehensive models for all major human and animal lineages of the *M*. *tuberculosis* complex will represent a challenge in the years to come. In addition, multiomics data are also desirable in order to capture the major regulatory layers^49^. The challenge will be to integrate all of them in a manner that can inform each other^50^ and to accommodate and predict the role of existing human and bacterial genetic diversity^40^. This a major challenge in the future for using systems biology approaches to prioritize targets for biomedical research in the *M*. *tuberculosis* complex.

## Materials and Methods

### Datasets and techniques used

The main microarray expression data sets were obtained from Rustad *et al*.^12^ (GEO accession number GSE59086). The ChIP-Seq data were obtained from Minsch *et al*.^13^. The TFOE-derived network used to compare with the TFOE network generated in this work was obtained from Rustad *et al*.^12^. The *phoP* mutant data were obtained from Solans *et al*.^37^ (GEO accession number GSE54241). The RNA-seq data from lineage 1 were obtained from Rose *et al*.^18^ (EBI ENA accession number ERP002122). The H37Rv RNA-seq data were obtained from Arnvig *et al*.^45^.

The R statistical language was used to perform all the analyses^51^, mainly the Bioconductor set of packages^52^. The workflow of the analyses performed in this work is summarized in Fig. 1.

### Model construction

The regulatory relationships used for model construction were obtained from Rustad *et al*.^12^ data set. We selected all the regulatory interactions with adjusted p-value <= 0.01 (Benjamini-Hochberg) regardless the fold-change in the expression values. In consequence, all the statistically significant regulatory influences (even the weak ones) were taken into account. From Minsch *et al*. (2015), we selected the physical bindings that demonstrated a regulatory effect on the level of gene expression of the target.

To select the TFs to be incorporated into gene-expression models we compared two different strategies. Firstly, for each gene expression model we selected those TFs that had a large influence on the expression response (Moderated t-test, adjusted p-value < 0.01 and fold-change > 2). Alternatively, we selected as relevant TFs all the genes showing regulatory influence (Moderated t-test, adjusted p-value < 0.01) without taking into account the intensity of the effect. The latter models were trimmed *a posteriori* following a backward step-wise methodology. In both cases, we evaluated whether the predicted values departed from random estimates by generating at least 20 random models incorporating some of the 200 TFs. To identify which of the two strategies predicted better the observed expression values in the Rustad *et al*. data set and to evaluate the strength of the prediction, we computed the correlation between predicted and real values of gene expression. We obtained an average correlation of 0.32 for the *a priori* approach and 0.71 for the *a posteriori* approach. As the differences in correlation estimates between both strategies were statistically significant (t-test, p-value < 0.01), we selected the latter method to construct the final models.

The following process was performed for each target gene in the TFOE- and the ChIP-Seq-derived models: 1. All the TFs affecting the gene were selected as regressors for the model. A TF is said to affect a gene if there is statistical evidence that the expression of the gene (moderated t-test adjusted p-value <= 0.01) changes when the TF is over-expressed following the procedure described in Rustad *et al*.^12^. In addition, the RNA polymerase alpha chain gene, *rpoA* (Rv3457c), and the sigma factor gene, *sigA* (Rv2703), were included as normalization factors. Interactions between TFs were also taken into account. The model structure^28^ is shown in equation (1).

1 $$y=a+\sum _{i={\rm{1}}}^{T}{b}_{i}{x}_{i}+\sum _{i={\rm{1}}}^{T}\sum _{j=i+{\rm{1}}}^{T}{c}_{ij}{x}_{i}{x}_{j}+d{x}_{sigA}+e{x}_{rpoA}+\varepsilon $$

where *y* is the target gene expression, *x*_*i*_ are the expression values of the selected TFs (from *i* = 1 to *T*), *a*, *b*, *d* and *e* are the linear coefficients in the regression model, *c* are the interaction coefficients, and *ε* is the error term.

2. A linear regression model with all the TFs selected as regressors and based on the previous structure was constructed. Next, the model was parameterized using microarray data from Rustad *et al*.^12^. This data set consists of 698 tiling microarrays, so that the model was fitted using 698 data points corresponding to expression values of the strains present in the data set.

3. The Bayesian Information Criterion (BIC) and the Akaike Information Criterion (AIC) associated to the model were calculated. To limit the overfitting error we used the BIC in the TFOE-derived models because it penalizes models with a large number of regressors^53^. In turn, the AIC was used when calculating models from the ChIP-Seq data set given the low number of regressors involved.

4. We sequentially eliminated from the model regressors whose removal led to the largest decrease in the BIC/AIC. So, we deleted from the model those terms whose removal had a minor or null contribution on the model’s performance. In biological terms, we filtered out the TFs that led to a minor or weak regulatory response in the target gene, in comparison with the rest of the TFs. The remaining TFs were retained and we returned to step 2. In case we did not observe a decrease in the BIC/AIC after the removal of any regressor, we considered that model as optimal for the corresponding gene.

5. A Fisher’s F-test was performed to check the null hypothesis that the retained regressors do not have predictive power^53^. P-values were adjusted to multiple testing by Benjamini and Hochberg false discovery rate (FDR)^54^ and all models with adjusted p-value >= 0.05 were rejected.

### Cross-validation of models

We checked the initial models obtained above in a 10-fold cross-validation.

For each gene:

1. The optimal model selected was parameterized using a random subset of the 90% TFOE data set as a training-set.

2. Next, the remaining 10% of the data set was used as a test-set to make predictions. A Fisher F-test was performed to check differences between residuals of the training set and the test set. Also, the Root Mean Squared Error (RMSE)^55^ was obtained when predicting over the test set.

3. Steps 1 and 2 were repeated 10 times (10-fold cross-validation)

We retained those models that showed no difference between predictions over the training set and the test set, by comparing the average adjusted p-value of the F-test over the 10 iterations (α >= 0.05). In some cases, we could not find differences between residuals but the squared error was high. In consequence, we also rejected models with RMSE > Q3 + 1.5 * IQR, as they were considered outliers of the RMSE distribution^56^.

### Comparisons to random models

We considered each TF of the data sets as a potential regulator. For each gene, we listed all the TFs that do not have a regulatory influence on it. From this list, we created 100 random subsets of TFs. The number of elements in each subset was equal to the number of real factors with regulatory influence on the corresponding gene. With this random subset of TFs, we followed the steps described above to create the random models. Also, the 10-fold cross-validation was performed for each random model.

For each model, a Welch’s t-test was performed to compare the distribution of p-values from the 10-fold cross-validation of the real model versus the random ones. P-values from Welch tests were adjusted by Storey’s method^57^. Tests showing a pFDR <= 0.01 were accepted as having a better fit than random models. Also, the RMSE distributions of random models were tested versus the real ones by means of a Welch’s t-test, correcting the p-values with Storey’s method. Tests showing a pFDR <= 0.05 were accepted.

### Comparison to a different TFOEs dataset

To check the ability of the models to perform accurate predictions over different datasets, we used a TFOE dataset similar to the one used to built the models but derived only from 50 TFs construct strains^28^. As gene expression values can have very different ranges it is not advisable to compare the numeric values directly. Predictive models are uncertain when predicting in a range of values different to the ones used to train them^53^. So, to compare both data sets we used the expression value of *dnaA* to normalize both the predicted values from the models and the absolute expression values from the experiments by^28^ and^12^. Pearson’s correlation was calculated between predicted and observed fold-changes.

### Evolutionary conservation of TFs within the MTBC

We have analyzed an available data set of natural polymorphisms in 219 representative strains of the complex, encompassing all known lineages and geographic distribution of the species^15^. All the genomic variants were called using the MTBC most likely ancestral state as a reference to ensure calling derived strains^58^. A custom script was used to search for TFs with at least 25% of their gene length deleted. A manual inspection was performed for the detected TF deletions to filter false positives and mapping errors. Also, single nucleotide polymorphisms (SNPs) leading to stop-codon gains or losses, and point mutations affecting TF regulatory regions^34^ were extracted from this dataset. The terminology used to classify the TSSs follows that of Cortes *et al*.^34^.

We defined the regulatory sub-network associated to each TF as that defined by the one-step distance nodes to the TFOE network. We studied for each sub-network the enrichment in certain functional categories as defined by the GO classification^59^. The Gene Set Enrichment (GSE) analysis was carried out using a combination of the BINGO tool^60^ and the Cytoscape software for visualization^61^. The enrichment test identifies the most abundant GO terms in the sub-network compared to all the possible terms present in the complete annotation, using a hypergeometric test (sampling without replacement).

### RNA-seq analysis

Expression data from H37Rv and lineage 1 strains were obtained from Rose *et al*.^18^. The differential expression analysis was performed using the DESeq2 package^62^. Differentially expressed genes were those with an adjusted p-value of 0.05. Differential expression analysis was performed over H37Rv versus all lineage 1 strains to test the differences in those genes regulated by Rv2788 (*sirR*). In addition, we specifically analyzed differentially expressed genes between strains T83 (lineage 1) and H37Rv (lineage 4).

### Predicting the impact of genetic polymorphisms

To predict the impact of the genetic background on the transcriptional landscape of the *M*. *tuberculosis* complex we chose a lineage 1 strain, namely T83. We have identified two genetic mutations in lineage 1 which likely have a major impact on the functionality of TFs. One of the mutations corresponds to a deletion affecting TF Rv1985c whereas the other is in an early stop codon in TF Rv2788. To simulate a transcriptional landscape for lineage 1, the expression values of both TFs were set to the minimum value found in the training dataset because standard regression models are only valid to make predictions in the same data range used to parameterize the model^53^. Gene expression values predicted for T83 were compared to those obtained from H37Rv expression models. We performed a Welch’s t-test over the expression values. P-values were adjusted by Storey’s method. A pFDR < 0.05 was considered for accepting the difference between models as significant. For the genes that showed differential expression, we calculated the log2 fold-change between H37Rv and T83. We compared these values with those obtained from the RNA-seq analysis. For a qualitative approach, we checked whether changes in expression values had the same sign (positive for induction and negative for repression). We constructed a 2 × 2 matrix with the predicted effect vs the measured effect and a Cohen’s kappa test was performed over this matrix to check the agreement between predicted and real data. To test the quantitative accuracy of the models we selected those genes that showed differential expression in the RNA-seq data set (adjusted p-value < 0.05) and in the predicted expression to calculate Pearson correlation.

Similarly, to make predictions on a *phoP* mutant in a H37Rv background we set its expression to the minimum value found in the training data set for this TF. The analysis was performed following the steps described above. To analyze how accurately the models reflect fold-changes in experimental data, we used an RNA-seq data from a *phoP* mutant and H37Rv^37^ as explained in the previous section. We compared the log2-based fold-changes between the predictive models and experimental data comparisons.

To compare the ChIP-Seq coverages in the different cases, we obtained raw data from the wild-type strain and the *phoP* mutant from Solans *et al*.^37^. We also downloaded ChIP-Seq data from the overexpression experiment of *phoP* (Rv0757_B167) from the MTB network portal^13^. The circular diagram was constructed with the Circos tool^63^ and the values from the regulatory influence were extracted from the TFOE data set.

### Data Availability Statement

The expression datasets analyzed during the current study are available at GEO repository with accession numbers GSE59086, GSE54241; EBI ENA repository under accession number ERP002122. All data generated during the current study are available from the corresponding author on reasonable request.

## Electronic supplementary material

Supplementary Figures 1–4

Supplementary Tables 1–6
