# Supplementary material for: Gene expression models based on a reference laboratory strain are poor predictors of Mycobacterium tuberculosis complex transcriptional diversity
Source: Sci Rep. 2018 Feb 28;8:3813. doi: 10.1038/s41598-018-22237-5 (PMC5830583; doi:10.1038/s41598-018-22237-5)
Supplement: Supplementary file 1 — Supplementary Figures 1–4 [file 41598_2018_22237_MOESM1_ESM.pdf]

Gene expression models based on a reference laboratory strain are poor predictors of *Mycobacterium tuberculosis* complex transcriptional diversity

Álvaro Chiner-Oms[1], Fernando González-Candelas[1,2] and Iñaki Comas[2,3*]

[1]Unidad Mixta "Infección y Salud Pública" FISABIO-CSISP/Universidad de Valencia, Instituto de Biología Integrativa de Sistemas, Valencia, Spain.

[2]CIBER en Epidemiología y Salud Pública, Valencia, Spain.

[3]Instituto de Biomedicina, IBV-CSIC, Valencia, Spain.

* Corresponding author:

E-mail: icomas@ibv.csic.es

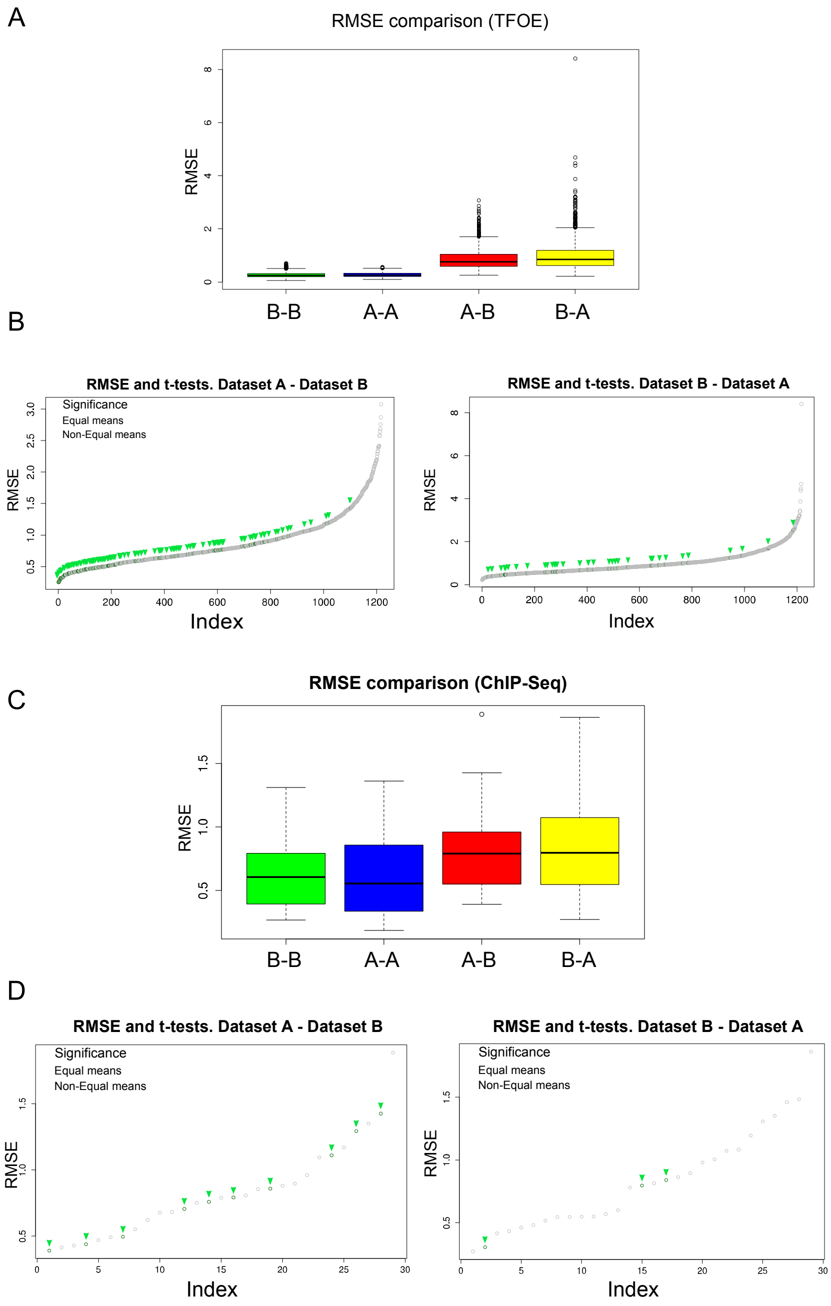

Supplementary Figure S1. Evaluation of accuracy and comparison of model's behavior between different datasets. The main goal of the models is to make predictions under different conditions and with several data sources. Therefore, apart from training the models with the same dataset used to calculate the models and the regulatory networks we trained them with the other analogous dataset. In this figure, dataset A is the one obtained from Rustad *et al*, and dataset B from Galagan *et al*. (A) Root mean squared error comparison (RMSE) for the models obtained from TFOE data. Values when training and testing with dataset B (green), training and testing with dataset A (blue), training with dataset A and testing with dataset B (red) and training with dataset B

and testing with dataset A (yellow). (B) Plot showing RMSE values for TFOE derived models. Index refers to the list of models sorted by RMSE. The green arrows mark those models having no differences between predicted and measured mean expression. The left plot shows the case of training with dataset A and testing with dataset B while the right plot shows the reverse case. In the left plot, 128 genes show no differences between real and predicted values in terms of equaliy of means while in the right plot 33 genes show no statistical differences (pFDR ≤ 0.01). (C) RMSE comparison for the models obtained from ChIP-Seq data. Values when training and testing with dataset B (green), training and testing with dataset A (blue), training with dataset A and testing with dataset B (red) and training with dataset B and testing with dataset A (yellow). (D) Plot showing RMSE values for ChIP-Seq derived models. Index refers to the list of models sorted by RMSE. The green arrows mark those models having no differences between predicted and measured mean expression. The left plot shows the case of training with dataset B and testing with dataset A while the right plot shows the reverse case. In the left plot, 10 genes show no differences between real and predicted values in terms of equality of means while in the right plot only 3 genes show no statistical differences (pFDR ≤ 0.01).

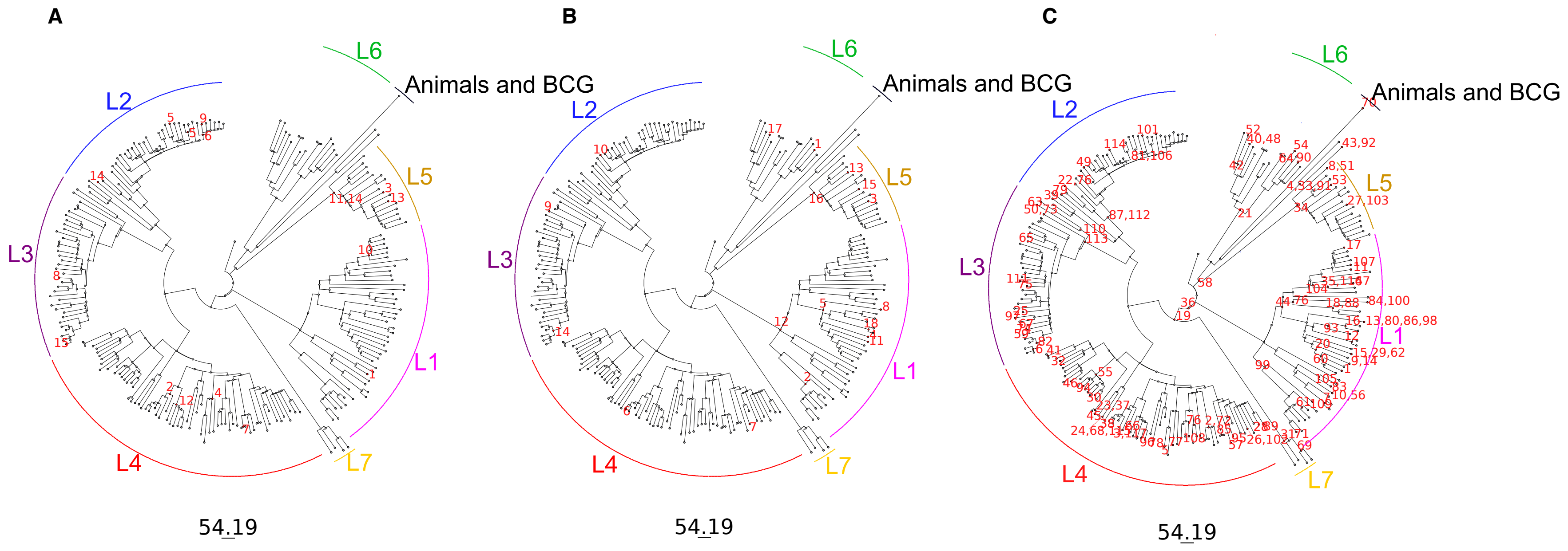

Supplementary Figure S2. Mutations affecting Tfs in the MTBC phylogeny comprising the seven major lineages. The figure represents the number of TFs missing or potentially affected in their regulatory functions in one or more clinical strains from the Comas *et al.* MTBC reference dataset (n = 219 strains). The mutations affecting a TF are mapped to the corresponding internal/external node of the Comas *et al*. 2013 phylogeny. Each panel shows the same phylogeny and the mutations affecting a TF are mapped to the corresponding branch in the tree and highlighted in red. Label numbers correspond to entries in Supplementary Table S3, Supplementary Table S4 and Supplementary Table S5. The mutations considered are either partial or complete deletions of the TF (A) (Supplementary Table S3), single point mutations leading to gain or loss of stop codons (B) (Supplementary Table S4) and single point mutations affecting the regulatory region of a TF (C) (Supplementary Table S5).

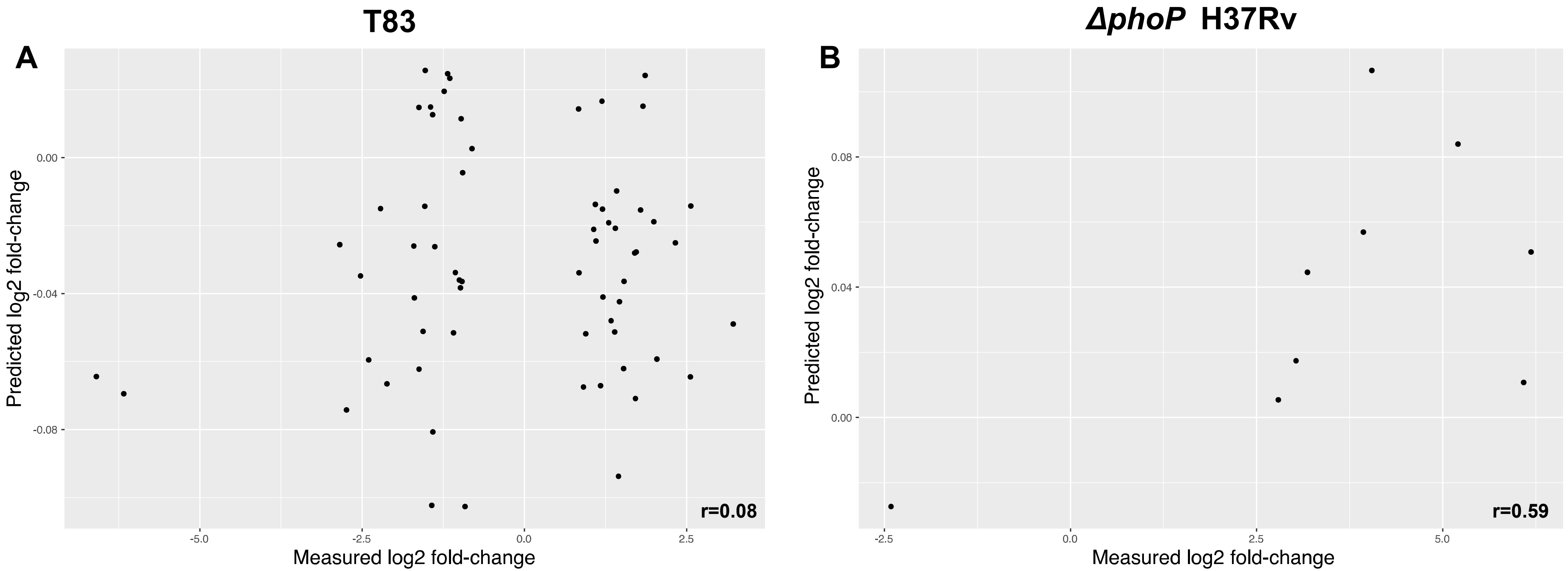

Supplementary Figure S3. Comparison between experimental and predicted fold-changes. A) The x-axis corresponds to the measured log2 fold-change in gene expression between H37Rv and the T83 strain in Rose *et al.* (2013). The y-axis corresponds to the predicted fold-changes calculated with the predictive models obtained in this work. B) The x-axis corresponds to the measured log2 fold-change in gene expression between the wild-type strain and the *ΔphoP* strain in Solans *et al.* (2014). The y-axis corresponds to the predicted fold-changes calculated with the predictive models.

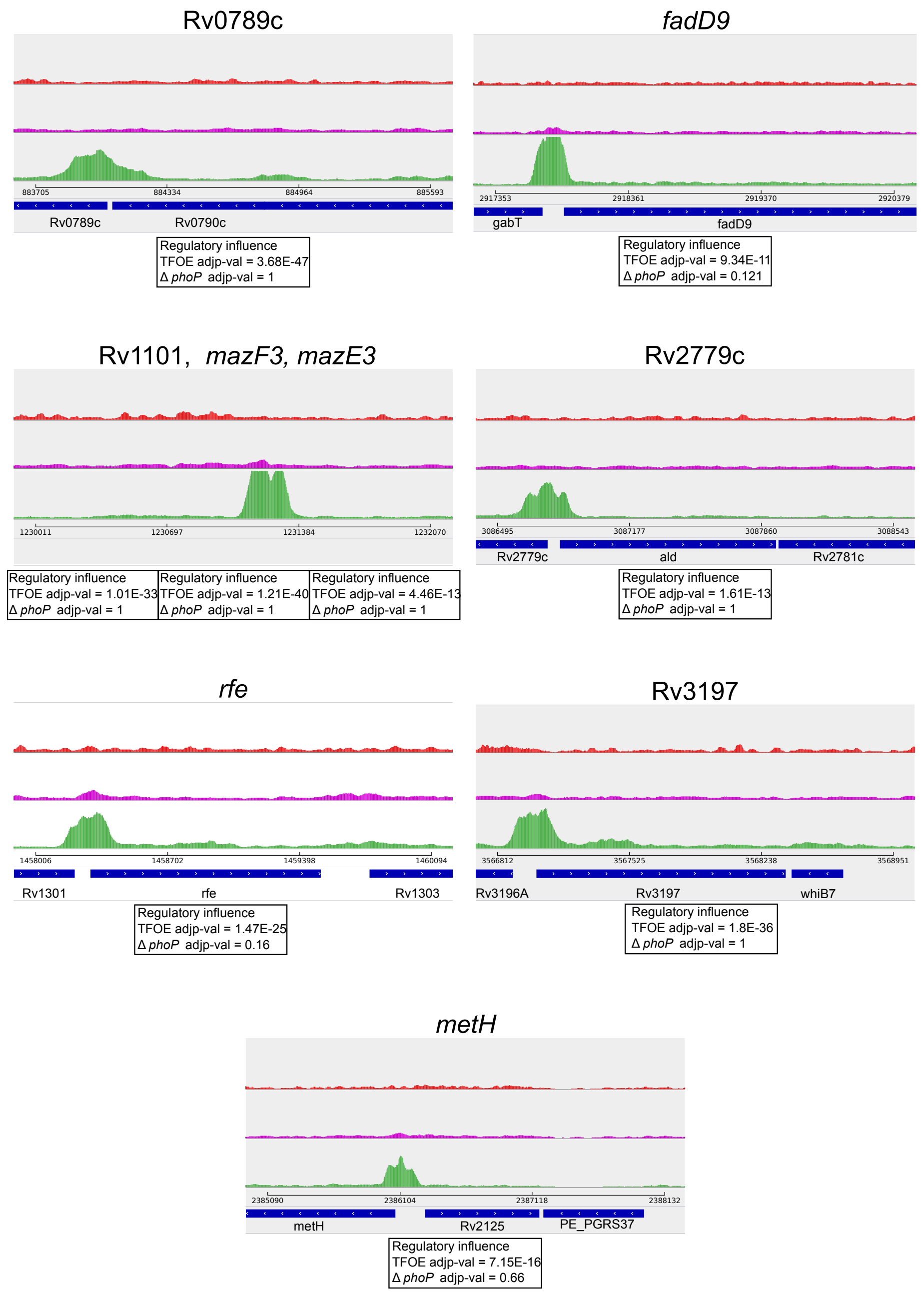

Supplementary Figure S4. Detailed information about 9 selected genes showing no agreement in their *phoP* regulatory signal depending on the experiment used to detected it. Detail of the 9 genes selected for which regulation is affected in *phoP* overexpression experiments but not in *phoP* knockout experiments. The red track corresponds to the level of PhoP binding in the regulatory region of the genes in a knockout strain. The magenta track corresponds to the level of binding in the wild type strain. The green track corresponds to level of binding in the *phoP* overexpressed strain. The impact on downstream transcriptional levels are shown as published previously for the TFOE data and for the *phoP* knockout data.
